# Watched or not: Overimitation in dogs under different attentional states

**DOI:** 10.3758/s13420-024-00635-2

**Published:** 2024-09-26

**Authors:** Louise Mackie, Jeanne Trehorel, Ludwig Huber

**Affiliations:** Comparative Cognition, Messerli Research Institute, Department of Interdisciplinary Life Sciences, University of Veterinary Medicine Vienna, Veterinärplatz 1, 1210 Vienna, Austria

**Keywords:** Domestic dogs, Attentional states, Copying, Overimitation, Social learning

## Abstract

Domestic dogs (*Canis familiaris*) have been documented to ‘overimitate’ humans – a form of social learning – by copying their causally-irrelevant actions. It is suggested that this behaviour results from social, affiliative motivations. Dogs have also been known to behave differently when they are being watched (or not) by humans, such as by following commands better (or worse). In this study, we tested whether dogs’ copying behaviour would also be sensitive to their caregiver’s attentional states. The subject’s caregiver demonstrated irrelevant and relevant actions in the dot-touching overimitation task, then during trials the caregiver was either watching their dog or turned away. Our results revealed no difference in dogs’ irrelevant-action copying; however, we found that dogs approached the dots less per trial when their caregiver was watching them. Dogs also copied their caregiver’s leftward sliding of a door (to obtain a food reward) more accurately when they were being watched by their caregiver. Finally, dogs who copied the irrelevant action did so more often after obtaining their food reward, which supports that these dogs may have had two separate goals: a primary instrumental goal and a secondary social goal.

**Supplementary information:**

The online version contains supplementary material available at 10.3758/s13420-024-00635-2.

## Introduction

If a dog’s caregiver turns away or leaves a room, their dog may decide to take some food from their dinner plate. When it comes to obeying caregiver commands or cues, domestic dogs (*Canis familiaris*) behave differently according to human attentional states. For example, Call et al. (2003) found that dogs approached a forbidden piece of food more indirectly and obtained the food less often if they were being watched by a human than if the human was engaged in another activity, turned around, or left the room completely. Dogs were shown to also ‘steal’ more food in front of a human if the target area had low levels of light (Kaminski et al., 2013). Similarly, dogs have been shown to obey a ‘lie down’ command more successfully and for longer while being watched (Schwab & Huber, 2006). Even when a dog’s caregiver would show attentive ambiguity (looking away at nothing), dogs responded uncertainly to a ‘lie down’ command (Virányi et al., 2004). One's sensitivity to the attentional cues of others demonstrates that one understands what others can or cannot see. This is a useful skill for individuals who have a will to please or obey others, such as domestic dogs – a species that have been following cues of, and cooperating with, their closely bonded humans for thousands of years.

Learning to follow human commands and cues is something that dogs are particularly good at. Many dogs have, and have had, their own jobs in human society. From search and rescue to post-traumatic stress disorder (PTSD) support, dogs can perform well in tasks for and with human partners after proper training (Alexander et al., 2011; Maoz et al., 2021). And unlike non-human primates (chimpanzees), domestic dogs perform on a similar level to human children in some sociocognitive tasks, especially those that involve following the human gaze or point (MacLean et al., 2017). Dogs’ high level of commitment towards their human partners may explain their tendency to copy them too.

Given their human-directed sociocognitive capabilities, it comes as no surprise that dogs are able to copy humans. In do-as-I-do tasks, dogs can be trained to copy both familiar and novel actions of human demonstrators, such as throwing a bottle or circling another human (i.e., Fugazza & Miklosi, 2014; Huber et al., 2009; Topál et al., 2006). Other studies have explored the imitative tendencies of dogs, finding that dogs (i) match the actions of human demonstrators to solve novel tasks (for a review, see Kubinyi et al., 2009), (ii) match humans as puppies (Fugazza et al., 2018) and without food rewards (Fugazza et al., 2023), (iii) struggle to *not* copy their caregiver’s action in a bidirectional task (Range et al., 2010), and even (iv) copy novel human actions after a delay (Fugazza, Pogany, & Miklosi, 2016; Huber et al., 2014). It seems that dogs cannot ignore even their caregiver’s goal-irrelevant actions in a novel problem-solving task, as they were shown to copy their caregiver’s (unnecessary) touching of coloured dots on the wall (Huber et al., 2018). Other studies have demonstrated this seemingly irrational faithfulness to humans, where dogs were found to follow an (mis)informant with a false belief (Lonardo et al., 2021) and a communicative demonstrator (Kupán et al., 2011) towards an empty food container. Notably, however, dogs do not always follow misleading human cues – for example, from an inaccurate informant in Pelgrim et al.’s (2021) study or from the true-belief informant of Lonardo et al.’s (2021) study. Dogs can recognise intentions (i.e., Völter et al., 2023), so whether or not dogs follow misleading cues may depend on whether these cues appear deceitful or not. Someone with a true belief or inaccurate pointing may come across as deceitful and therefore untrustworthy.

The copying of another’s irrelevant actions was first documented in human children (Horner & Whiten, 2005), and was then called ‘overimitation’ by Lyons et al., (2007). However, Whiten et al. (2009) preferred to use the more neutral term ‘over-copying’ since overimitation studies do not typically examine the faithful replication of movement trajectories (e.g., Huber, 1998), which is often a necessary feature when defining *imitation*. Nonetheless, overimitation is the most common term in the literature. We henceforth use overimitation in the sense of general irrelevant-action copying or matching, when action causal-irrelevance is perceivably obvious (i.e., Lyons et al., 2007).

For humans, there is evidence that overimitation is influenced by social factors, such as the demonstrator’s characteristics, the presentation format of the demonstration, and the language used during the overimitation tasks (for a review, see Hoehl et al., 2019). One notable factor that influences human overimitation is the attentional states of the demonstrator. Stengelin et al. (2019) showed that children, from both Namibia and Germany, copied more (irrelevant) actions from a model who was watching them during their turn to operate the task’s puzzle box. Another study showed that children reduced their overimitation when a model turned away, but not when the model was watching them or had left the room (Marsh et al., 2019). Dogs are also sensitive to human attentional states in terms of obedience, but they have not (yet) been tested for this sensitivity in a social learning context.

It is suggested that dogs copy irrelevant actions for social, affiliative motivations. For example, dogs have proceeded to overimitate their caregiver more than a stranger (Huber et al., 2020), have obtained high overimitation scores alongside high caregiver-relationship scores (Huber et al., 2022), and have often overimitated after already achieving their instrumental (food) goal (Mackie & Huber, 2023). Dog puppies have also spontaneously copied human actions such as touching a box with the nose in the complete absence of food rewards, which would make these actions also causally irrelevant to an instrumental (food) goal (Fugazza et al., 2023). The task used in Huber et al.’s (2018, 2020, 2022) and Mackie and Huber’s (2023) studies was a dot-touching task for dogs, purposely designed to physically disconnect irrelevant and relevant actions, thus increasing causal transparency between these two actions. Often human-designed puzzle boxes have pseudo-instrumental irrelevant actions, meaning that the physical connectedness of these actions could easily be misinterpreted as causal relevance to obtaining the reward (i.e., Schleihauf & Hoehl, 2021). A disconnection would reduce the chances of overimitation occurring as a result of causal misunderstanding. So, the motivation for a dog to overimitate, by copying the touching of (at least) one or more irrelevant coloured dot(s) on the wall in Huber et al.’s (2018) task, is likely to have other (social) reasons behind it. But it is unclear whether these reasons are related to affiliation, to be like or associate with the bonded-caregiver, or perhaps an intention to please their *watchful *caregiver, someone who usually holds expectations that the dog should follow their commands (in this case, actions interpreted as cues).

As dogs have shown sensitivity to human attentional states in obedience tasks – tasks to follow human commands or cues – would they alter their overimitation behaviour if their caregiver is watching them or turned away? If so, then copying irrelevant (or relevant) actions may be related to the dog’s will to please their watchful caregiver (i.e., as in Schwab & Huber, 2006), rather than pure affiliation. Our study investigated dogs’ sensitivity to their caregiver’s attentional states in the context of the dot-touching overimitation task (from Huber et al., 2018). After the dog received their caregiver’s task demonstration (containing both irrelevant and relevant actions), we manipulated whether the caregiver would be watching them closely or turned away facing a wall during the dog’s task trial (four demonstrations and trials total). We scored each dog for their irrelevant-action copying accuracy (from 0 to 4) during these trials, where a score of two plus (touching at least one irrelevant dot) was considered overimitation. Thus, with a two-tailed hypothesis that dogs may be overimitating for either affiliation or simply following cues, we predicted that dogs would either have equally high irrelevant-copying accuracy scores between conditions (caregiver watching or caregiver turned away), *or* dogs would score higher when their caregiver was watching them. Additionally, if dogs are overimitating for non-instrumental (social) reasons, we also predicted that overimitation would more often occur *after* obtaining the reward, since this was found to be the case in Mackie and Huber (2023). Dogs may prioritise the attractive food reward in the dot-touching task to only later copy their caregiver’s irrelevant actions, especially if their overimitation has alternative social motivations. We did not make any predictions concerning any copying of the goal-relevant action, but we included the measurement and analysis of the dog’s relevant-action copying between our two conditions.

## Methods

### Ethics statement

**Fig. 1:**
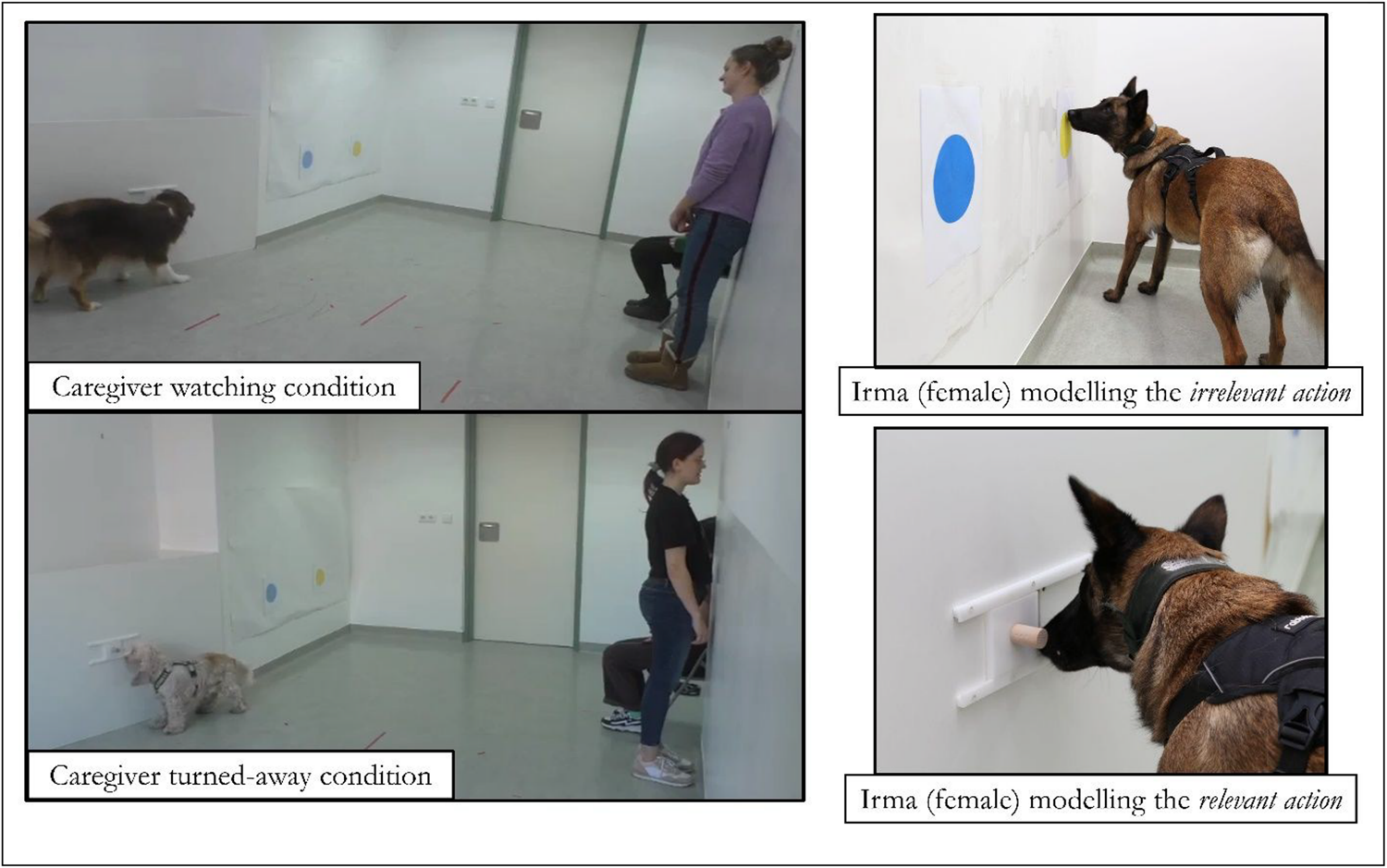
The dot-touching overimitation task. On the left, examples of the caregiver’s position in each condition (watching and turned-away). On the top right, a dog modelling the touching of the yellow dot with her nose to copy part of the *irrelevant action*. On the bottom right, a dog modelling the sliding open of the food chamber door to copy part of the *relevant action*

The ethics committee of the University of Veterinary Medicine Vienna approved this study and its procedures, in agreement with good scientific practice and national legislation guidelines (ref: ETK-173/10/2022). Written consent was obtained from the dogs’ caregivers, who participated voluntarily with their dogs and were told that they could withdraw from the study at any time. Extra informed consent was obtained from the caregivers who appear in the pictures in Fig. 1. Dogs engaged in a non-invasive problem-solving task to obtain familiar food rewards (i.e., pieces of sausage or caregiver-approved dog treats), and were together with their caregiver for the duration of the session at the Clever Dog Lab.

### Participants

Closely matching previous studies with the dot-touching task (e.g., Huber et al., 2018), the final sample size of this study was 63 family dogs (31 female, 32 male) after four dogs were pilots and 11 dogs were excluded (for technical issues, lack of motivation, attempted caregiver assistance in trials, such as pointing, or caregiver demonstration error/experimenter error). Therefore, the total number of dogs tested at the Clever Dog Lab for this study was 78. The first 15 dogs were randomly assigned to the two conditions, while the remaining 48 were assigned to balance the conditions for age, sex and breed. There were 33 dogs (16 females, 17 males) in the watching condition and 30 dogs (15 females, 15 males) in the turned-away condition. Mixed dogs represented the highest number of subjects (23 dogs), but no breed was over-represented in the final sample. See Online Supplementary Materials (OSM) for Participant List (which contains; dog age, breed, condition, obedience score, if they had reported training, task success, if they were an overimitator or not, and Clever Dog Lab experience).

Dogs and their caregivers were recruited through the Clever Dog Lab database and website, social media, and from dog parks. All dogs were required to be between 1 and 12 years old, food motivated, above 30 cm (to reach the apparatus door), and naïve to the dot-touching overimitation task.

### Design and procedure

#### Procedure

Caregivers arrived at the Clever Dog Lab with their dogs to first read and sign the study’s consent and data protection forms. During this time, caregivers were in the presence of the experimenter who could answer questions about the study and tell caregivers that they could withdraw at any time. Caregivers were not informed of the study’s hypotheses, or about overimitation in particular, until after the session was complete.

The session itself took place in the green room of the Clever Dog Lab (6.0 × 3.3 m) and lasted around 20 min. It was recorded on two video cameras (Panasonic HC-V777, www.panasonic.com) and one ceiling camera. Their outputs were framed together in an.mp4 video file for behavioural coding. The session contained four phases for each dog: a 1-min habituation phase in the room off leash, a 1-min obedience test, a warm-up cup-game, and lastly, four caregiver demonstrations and dog trials of the dot-touching overimitation task, in which we had one of the two manipulations of the caregiver’s attentional state (watching or turned away). On completion of the session, the dog was guided to the food reward if they were unsuccessful and the caregiver received an A4-sized study certificate for their participation.

#### Design

This study had a mixed design. We had four task trials, which were a within-subject measure, and two between-subject conditions, where the only difference was that of the attentional state of the caregiver during the dot-touching overimitation task. In the *watching condition*, the caregiver closely watched their dog during the four trials of the task, and in the *turned-away condition*, the caregiver turned away and faced the wall during the four trials of the task.

#### Obedience test

Sixty dogs experienced a mini-obedience test before the warm-up and the main task. The obedience test lasted 1 min and contained five commands (including sit and stay) from the caregiver in the absence of food rewards. This test was to calculate a score of command-following for each dog (Appendix A). However, due to large behavioural variance we did not extract reliable scoring from the obedience test to analyse (see *Behavioural coding*).

#### Warm-up cup-game

All dogs experienced a warm-up cup-game in attempt to raise the dog’s motivation to pay attention to the caregiver and to collect food rewards. The warm-up also ensured that the dog could (and was motivated to) retrieve food in preparation for the main task. The cup-game itself was a replication of Mackie and Huber’s (2023) and Huber et al.’s (2018) attention task. Three cups were placed upside-down, 60 cm apart, and 2 m in front of the dog (on a leash with the experimenter sitting behind). The caregiver kneeled behind the cups and placed a piece of kibble (or equivalent treat) under one of them for the dog to find. The dog was released by the experimenter and chose a cup by sniffing or touching it. If the first choice was correct, the dog was allowed to eat the reward. There were six rounds of the warm-up cup-game, with the food position alternating between the middle cup (three times), the left, the right, and the middle cup again.

#### The dot-touching overimitation task

Finally, all dogs participated in four trials of a novel problem-solving task to obtain a piece of sausage (or equivalent treat) – the dot-touching overimitation task (from Huber et al., 2018). The set-up of this task included: (i) two coloured dots (blue and yellow) printed on separate A4-sized sheets of paper and stuck to the wall (around 55 cm apart, 50 cm above the floor, and 130 cm to the left of the food chamber), (ii) a wooden panel (150 × 100 cm) containing a food chamber (6 × 7 cm) hidden behind a sliding door (10 × 9 cm) mounted in the middle of the panel (50 cm above the floor), and (iii) a chair on the opposite side of the room for the experimenter to sit on with the dog next to them (around 200 cm away from the dots and wooden panel).

Each trial began with a caregiver task-demonstration. This was taught to the caregiver by the experimenter through a video example, then the caregiver was asked to practice it before beginning the task. When ready, the experimenter, caregiver and dog (on a leash) entered the testing room. The experimenter sat in the chair with the dog by her side (in a position to view the demonstration). The caregiver (standing next to the dog and experimenter) then walked towards the opposite wall and positioned on his/her hands and knees to demonstrate the task’s actions in a dog-like manner. To demonstrate the *causally-irrelevant action*, he/she looked at the dog (or called their name if necessary) to ensure the dog was paying attention, and touched the blue dot, then the yellow dot, with his/her nose. Then to demonstrate the *causally-relevant action*, he/she moved to the wooden panel, got on his/her hands and knees, and slid the door open to the left with his/her nose (while maintaining the dog’s attention). He/she took the food from the chamber to show the dog, then placed it back and slid the door closed again (hiding this with his/her body). Afterwards, the caregiver returned to stand by the experimenter and dog (Fig. 1).

Depending on the condition, the caregiver either stood watching the dog or turned away to face a wall for the four trials. The dog was released to begin their 1-min task trial. During the trial, the caregiver was instructed to stand still and not interrupt or help the dog in any way, while the experimenter did the same. Dogs were excluded from the study if the caregiver attempted to influence the dog during trials, such as by pointing at the food chamber. Figure 1 displays an example of the caregiver’s standardised position in each condition and examples of the irrelevant and relevant actions.

### Behavioural coding and data analysis

#### Behavioural coding

Video recordings were uploaded to an in-house Loopy (http://loopb.io, loopbio gmbh, Vienna, Austria) server for storage and behavioural coding. Dog behaviours of interest were the irrelevant- and relevant-action scores from the dot-touching overimitation task (see Table 1 for behaviour descriptions). We also coded the irrelevant-action copying’s (overimitation’s) timing with regards to the goal – whether dogs touched at least one dot before or after obtaining their food reward. Command-following scores were coded from the obedience test, with the score representing the number of commands followed, with a maximum score of 5 (see Appendix A for details).

**Table 1:** Irrelevant- and relevant-action score behavioural descriptions for coding

Action score	Irrelevant action description	Relevant action description
0	No approaching of the wall with dots	No approaching of the food chamber’s panel
1	Approaching of the wall with dots: dog walked/smelled directly in front of the wall with dots	Approaching of the food chamber’s panel: dog walked/smelled directly in front of the panel
2	Touching of one of the dots with the nose	Touching of the food chamber area of the panel with the nose
3	Touching of both dots with the nose, in the wrong order (yellow, blue)	Pushing open of door with nose, in the wrong direction (towards right) to obtain the food reward
4	Touching of both dots with the nose, in the correct order (blue, yellow)	Pushing open of door with door, in the correct direction (towards left) to obtain the food reward

To examine the approach behaviour separately from the irrelevant-action copying, we decided to conduct secondary, exploratory analyses in a binary manner for *overimitation* (yes – touched a dot, no – did not touch any dot) and *approach* (of the dots) behaviour (yes – approached dots, no – did not approach dots). Mackie and Huber (2023) had varying findings for these two types of behaviours; with no effect of trial for their overimitation scores but a decrease per trial for approaching the dots. We therefore recoded the irrelevant-action scores (ordinal, 0–4) into an overimitation score (binary, yes or no) and an approach score (binary, yes or no) for two binomial mixed models. Overimitation was scored as yes if the dog touched at least one dot in its trial and no if it did not. Approach was coded binomially for whether or not the dog approached the wall with the dots in its trial, by walking or smelling directly in front of either one or both of them (0 for no, 1 for yes).

The experimenter (JT) coded all of the 63 dog videos for these behaviours. Additionally, 20% (13 randomly selected videos) were coded by KG, an external and naïve coder, to analyse the coding reliability. KG received a scoring guide (resembling Table 1) and cropped videos, which contained only the obedience test and the four 1-min trials of the dot-touching overimitation task (without their demonstrations). The inter-coder agreement (weighted Cohen’s Kappa) was almost perfect for the irrelevant-action scores (0.98), perfect for the relevant-action scores and irrelevant-action timing (1.0), but the command-following scores only had substantial agreement (0.74).

#### Data analysis

The data were analysed using the software R Studio. To examine whether the attentional state of caregivers affected the dogs’ performance during the overimitation task, we fitted an ordinal (i.e., cumulative logit link) mixed model. We built two models, one with the accuracy score of the irrelevant action (OI_score) and one using the accuracy score of the relevant action (REL_score) as response variables (Table 1). Both models were identical and included condition (turned-away/watching) as the test predictor of interest, along with trial number (1, 2, 3, 4), age squared, and sex (male/female) as control predictors. We also included subject as a random intercept effect to account for repeated measurements of the same individuals, and trial within subject as a random slope effect (Barr et al., 2013; Schielzeth & Forstmeier, 2009). The obedience score was not included in the ordinal mixed models, as it proved difficult to score reliably given the variation of dog behaviours during the obedience test. This measure also would have required us to subset the data. Additionally, two dogs’ relevant action scores were removed for experimenter error and one dog’s fourth trial was removed for technical issues. We fitted the models in R (Version 4.2.0, R Core Team, 2022) using the clmm function of the package ordinal (Version 2020.8–22). Prior to fitting the model we z.transformed the covariates trial number and age, to ease model convergence and achieve easier interpretable model coefficients (Schielzeth, 2010). The full ordinal mixed models are shown below.

Full ordinal model (irrelevant action):

$$\text{IRR}\_\text{Score }(0-4)\sim\text{ Condition }+\text{ z}.\text{trial }+\text{ z}.\text{age }+\text{ I}(\text{z}.\text{age}^\text{2})+\text{ sex }+(1+\text{ z}.\text{trial }\vert\text{ subject})$$

Full ordinal model (relevant action):

$$\text{REL}\_\text{Score }(0-4)\sim\text{ Condition }+\text{ z}.\text{trial }+\text{ z}.\text{age }+\text{ I}(\text{z}.\text{age}^\text{2})+\text{ sex }+(1+\text{ z}.\text{trial }\vert\text{ subject})$$

We verified the absence of collinearity by calculating the Variance Inflation Factor (VIF) for a corresponding linear mixed model using the R package ‘car’ version 3.0–12 (Fox & Weisberg, 2019). This revealed that collinearity was not an issue (max VIF REL_score: 1.21; max VIF OI_score: 1.16). After fitting the model, we confirmed that the model assumptions of proportional odds were not violated by dichotomizing the scoring as at least 1, at least 2, at least 3, and at least 4, fitting logistic models with the obtained response variables, and inspecting the model estimates. These varied only a little, suggesting that the assumption was not strongly violated. We assessed model stability with respect to the model estimates by comparing the estimates from the model including all data with estimates obtained from models in which individuals were excluded one at a time (Nieuwenhuis et al., 2012). This revealed that the models were of good stability. We determined the significance of individual predictors by dropping them from the full model, one at a time, and comparing the resulting model with the full model. For model comparisons, we used a likelihood ratio test (Dobson and Barnett, 2018). We calculated 95% confidence intervals for the model estimates and fitted values by applying the function ‘bootMer’ of the package ‘lme4’, applying 1,000 parametric bootstraps, or the ‘confint’ function in R depending on the model complexity. We also used Nakagawa and Schielzeth’s (2013) R-squared from the ‘r2_nakagawa’ function in R to obtain marginal (fixed effects) and conditional (total model) R-squared values for effect sizes.

By replicating the above analysis method, we conducted two additional exploratory analyses on overimitation and approach (of the dots) behaviour. These models also included a two-way interaction between condition and z.trial, which, if non-significant, would be removed to interpret the lower term effects (Underwood, 1997). Overimitation scores (yes/no) and approach scores (yes/no) were analysed in two binomial mixed models:

Full binomial mixed model (overimitation):

$$\text{OI}\_\text{Score }(\text{yes}/\text{no})\sim\text{Condition}^\ast\text{ z}.\text{trial }+\text{ z}.\text{age }+\text{ I}(\text{z}.\text{age}^\text{2})+\text{ sex }+(1+\text{ z}.\text{trial }\vert\text{ subject})$$

Full binomial mixed model (approach):

$$\text{IRR}\_\text{Approach }(\text{yes}/\text{no})\sim\text{Condition}^\ast\text{ z}.\text{trial }+\text{ z}.\text{age }+\text{ I}(\text{z}.\text{age}^\text{2})+\text{ sex }+(1+\text{ z}.\text{trial }\vert\text{ subject})$$

Regarding the timing of overimitation (touching at least one irrelevant dot), whether it was before or after the goal, we conducted an exact binomial test in R with a 0.5 proportion for the number of trials where dogs both overimitated and obtained the reward (N = 29), as in Mackie and Huber (2023).

Data and R-scripts are available on the Open Science Framework (OSF) through the following link: https://osf.io/dkbsg/

## Results

### Descriptives

Overall, there were 24 overimitators (dogs who touched at least one dot in one trial) out of 63 dogs. Seven of those dogs overimitated repeatedly (in more than one trial). There were 29 (out of 251 total) trials where dogs *both* touched at least one dot and obtained the reward. Overimitator age varied greatly, between 1 and 9 years old, and there were 11 males and 13 females. Of the 13 dogs who were reported to have any kind of training in the database, six were overimitators and seven were not. Additionally, 33 dogs successfully obtained the food reward from behind the sliding door at least once in their trials (16 female, 17 male). Six (out of 13) dogs with reported training history obtained the food reward at least once. Regarding age, the five (out of five) oldest dogs (9 to 12 years old) and nine (out of 16) of the 1-year-old dogs obtained the food reward at least once (see Participant List in OSM).

### Irrelevant-action scores and attentional states

**Fig. 2:**
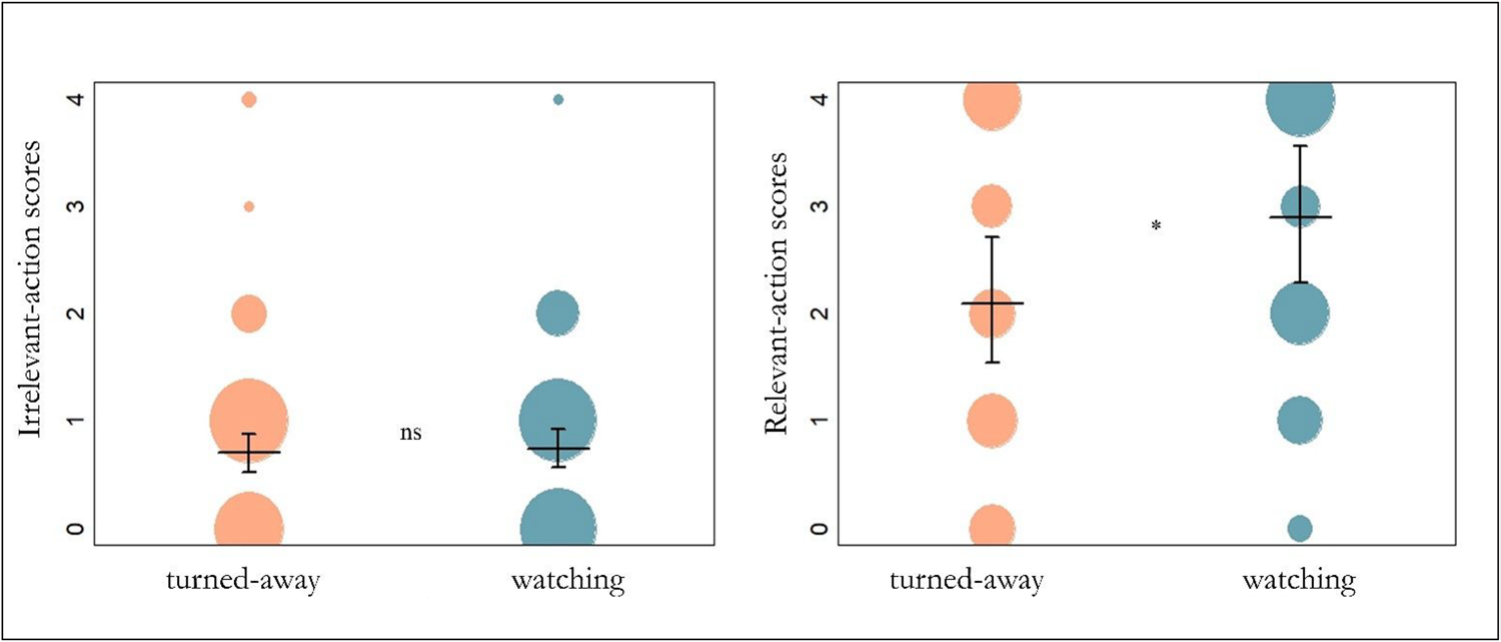
Results of the two ordinal mixed models for caregivers’ attentional states and dogs’ action scores. The left plot shows the number of times that dogs scored 0–4 for the irrelevant action in each condition (turned-away (orange)/watching (blue)); “ns” = not significant in the model. The right plot shows the number of times that dogs scored 0–4 for the relevant action in each condition (turned-away / watching); * = a significant effect in the model (*p* < .05). The vertical lines represent each model’s confidence intervals while the horizontal lines are each model’s fitted values. The circle size represents the number of times the corresponding score was scored by a dog

The full-null model comparison for irrelevant-action scores was non-significant (χ^2^ = 0.066, df = 1, p = 0.797), meaning that the caregivers’ attentional states (turned-away or watching) had no effect on the dogs’ accuracy scores for irrelevant actions (Fig. 2). The model revealed no significant effects for condition, age, age squared, or sex on irrelevant-action scores. However, there was a significant effect of trial (χ^2^ = 6.084, df = 1, p = 0.014), in that dogs’ irrelevant-action scores decreased as trial numbers increased. Nakagawa’s marginal *R*^2^ value was 0.036 and the conditional *R*^2^ value was 0.277, thereby 3.6% of the irrelevant-action scores’ variability could be accounted for by our fixed effects and 27.7% by the total model. Results of the full ordinal mixed model for irrelevant-action scores are available in Appendix B.

### Overimitation and attentional states

The full-null model comparison for overimitation scores (yes/no) was non-significant for the binomial mixed model (χ^2^ = 1.129, df = 3, p = 0.77), meaning that the caregivers’ attentional states (turned-away or watching) had no effect on the dogs’ overimitation. This model revealed no significant effects for condition, age, age squared, trial or sex on overimitation behaviour. Nakagawa’s marginal *R*^2^ value was 0.034 and the conditional *R*^2^ value was 0.331, thereby 3.4% of the overimitation scores’ variability could be accounted for by our fixed effects and 33.1% by the total model. Results of the full binomial mixed model for overimitation are available in Appendix C.

### Approaching dots and attentional states

**Fig. 3:**
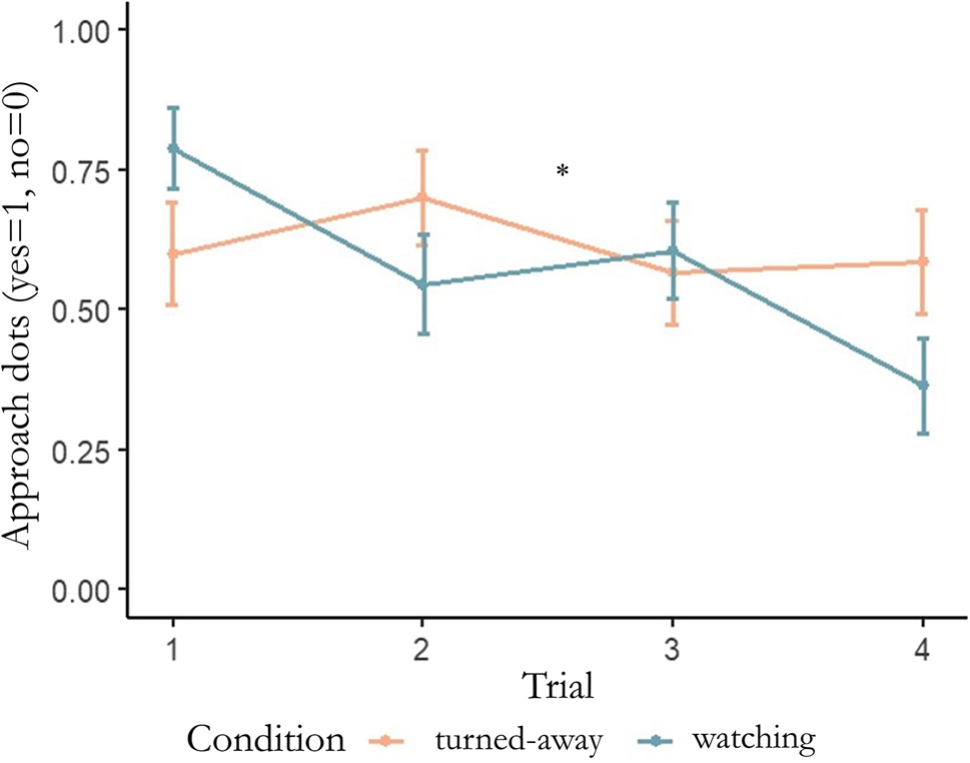
The binomial mixed model’s significant interaction between condition and trial for approach (of the dots), visualised with means (Appendix D). The trial means of condition “ownerfacewall” are represented along an orange line and ‘ownerwatchdog’ along a blue line

The full-null model comparison for approach (of the dots) behaviour was significant in our binomial mixed model (χ^2^ = 12.665, df = 3, p = 0.005), meaning that the caregivers’ attentional states (turned-away or watching) had an effect. The binomial mixed model for approach behaviour revealed a significant interaction between condition and trial (χ^2^ = 4.623, df = 1, p = 0.034), where approaching behaviour decreased per trial only when the caregiver was watching the dog (Fig. 3). There were no significant effects of age, age squared, trial or sex. Nakagawa’s marginal *R*^2^ value was 0.107, thereby 10.7% of the approach behaviour’s variability could be accounted for by our fixed effects. Results of the full binomial mixed model for approach are available in Table 2.

**Table 2:** Results of the significant binomial mixed model for the irrelevant-action approach (of the dots) behaviour (yes/no)

Term (effect)	Model estimate	Std. error	Z value	df	P value	Min	Max
(intercept)	0.581	0.346	1.679	1	0.093	0.446	0.708
Condition_watching	0.027	0.416	0.066	1	0.947	-0.092	0.189
z.trial^1^	-0.091	0.207	-0.440	1	0.660	-0.178	-0.014
z.age^1^	-0.186	0.278	-0.670	1	0.503	-0.268	-0.089
I(z.age^2)^1^	-0.638	0.167	-0.669	1	0.504	-0.147	-0.066
Condition_watching*z.trial	**-0.638**	**0.300**	**-2.123**	**1**	**0.034**	**-0.740**	**-0.561**

Z value represents the z statistic, df represents degrees of freedom, p value indicates the significance level (bold rows are significant; *p* < .05), and min. and max. are the minimum and maximum model stability estimates, respectively

1 Trial number and age were z-transformed to a mean of zero and a standard deviation of one, mean (SD) of trial number was 2.49 (1.12), mean (SD) of age was 3.96 (2.89)

### Overimitation timing (before goal/after goal)

There were 29 trials where dogs both overimitated (touched at least one dot) and obtained the reward (opened the door and ate the food) in a trial. In 22/29 of these trials, dogs overimitated after already achieving the goal (touched at least one dot after eating the food reward). In 7/29 of these trials dogs overimitated before achieving the goal. Compared to a 50:50 chance, the proportion of dogs overimitating afterwards was much larger than the proportion of dogs overimitating beforehand (exact binomial test; *N* = 29, p = 0.008).

### Relevant-action scores and attentional states

The full-null model comparison for the copying accuracy of relevant actions was significant (χ^2^ = 4.575, df = 1, p = 0.03), meaning that the caregivers’ attentional states (turned-away/watching) had an effect on relevant-action copying scores (Fig. 2). The model revealed that dogs copied the relevant action significantly better when they were being watched (χ^2^ = 5.753, df = 1, p = 0.017), that dogs copied the relevant action significantly better per trial (χ^2^ = 8.167, df = 1, p = 0.004), and younger and older dogs copied the relevant action better than middle-aged dogs (χ^2^ = 4.575, df = 1, p = 0.03). The model had no significant effect of age and sex (see Table 3 for full ordinal mixed model results of relevant-action copying). Nakagawa’s marginal *R*^2^ value was 0.138 and the conditional *R*^2^ value was 0.841, thereby 13.8% of the relevant-action scores’ variability could be accounted for by our fixed effects and 84.1% by the total model.

**Table 3:** Results of the significant ordinal mixed model for relevant-action behavioural scores (0–4)

Term (effect)	Model estimate	Std. error	Lower cl	Upper cl	χ2	df	P value	Min	Max
0|1	-3.433	1.012	-6.066	-1.208			^(3)^	-3.852	-2.986
1|2	-0.874	0.960	-3.138	1.286			^(3)^	-1.256	-0.389
2|3	2.045	0.986	-0.111	4.282			^(3)^	1.700	2.597
3|4	3.697	1.038	1.453	6.047			^(3)^	3.371	4.215
**Condition_watching**^**1**^	**1.928**	**0.915**	**-0.220**	**4.396**	**4.575**	**1**	**0.033**	**1.494**	**2.291**
**z.trial**^**2**^	**0.785**	**0.302**	**0.265**	**1.440**	**8.167**	**1**	**0.004**	**0.662**	**0.848**
z.age^**2**^	-0.562	0.596	-2.118	0.816	0.895	1	0.344	-0.841	-0.292
**I(z.age^2)**^**2**^	**0.932**	**0.398**	**0.081**	**2.124**	**5.753**	**1**	**0.017**	**0.768**	**1.067**
sexM^**1**^	-0.306	0.837	-2.271	1.561	0.134	1	0.715	-0.577	-0.038

cl represents the confidence interval limit, χ^2^ represents the chi-square statistic, df represents degrees of freedom, p value indicates the significance level (bold rows are significant; *p* < .05), and min. and max. are the minimum and maximum model stability estimates, respectively

1 Condition and sex were dummy coded with ‘Condition_turned-away’ and ‘female’ being the reference categories, respectively

2 Trial number and age were z-transformed to a mean of zero and a standard deviation of one, mean (SD) of trial number was 2.49 (1.12), mean (SD) of age was 3.96 (2.89)

3 Not indicated because of having a very limited interpretation

## Discussion

In summary, dogs who were being watched by their caregivers had significantly higher relevant-action scores, but not irrelevant-action scores, than dogs whose caregivers were turned away during trials. Additionally, younger and older dogs performed better than middle-aged dogs with respect to their relevant-action scoring. For each trial, dogs scored higher and higher for the relevant-action, but lower and lower for the irrelevant-action. However, after we separated overimitation scores (dogs touching at least one dot) from scores of approaching the dots, we found no trial effect for overimitation and an interaction between condition and trial for the approach behaviour. Dogs approached the wall with the dots less per trial when being watched, yet no differently over trials when their owner was turned away. Finally, for overimitation timing, dogs overimitated more often after (rather than before) achieving the food goal in trials where they both touched at least one dot and got the reward. Overall, the number of dogs considered to be overimitators in this study was slightly lower than in previous studies using the dot-touching task (i.e., Huber et al., 2018; Mackie & Huber, 2023).

Firstly, we found an interaction between caregiver attentional states and trials for dogs approaching the dots. By conducting two separate analyses on overimitation and approach scores, we found that the effect of trial was lost for touching at least one dot, but remained for approaching the dots (replicating Mackie and Huber’s (2023) findings). The fact that dogs have now repeatedly performed differently in these behaviours reiterates a distinction between dogs’ lowest level of irrelevant-action scores, their exploration-like approach behaviour, and their overimitation in this task. Results showed that approaching the dots decreased over multiple trials when the caregiver was attentive, but not when she/he was facing the wall. There may have been an initial expectation that approaching the dots would elicit a response from the watchful caregiver. During trials though, caregivers did not interact with or react to their dogs, and dogs likely learned this over trials. Dogs may have behaved ‘obediently’ or ‘pleasingly’ towards their watchful caregiver by approaching the dots at first (the location of the caregiver’s irrelevant demonstration), but were discouraged over time without any reinforcement from their caregiver and with the discovery of the food-reward location. Alternatively, secure-base effects are found in dog-caregiver bonds – for example, like children, dogs explore new rooms, play with strangers, and play more independently in the presence (rather than absence) of their caregivers (Palmer & Custance, 2008) – so dog exploration-like approaching in our task could have been initially facilitated by the secure-base effect from an attentive caregiver.

Secondly, we found that overimitation behaviour occurred independently of caregiver attentional states, unlike that found in children (Marsh et al., 2019; Stengelin et al., 2019), and unlike dog obedience behaviour (Call et al., 2003; Schwab & Huber, 2006; Virányi et al., 2004). For dogs who overimitated, the motivation to copy and match the caregiver’s irrelevant demonstration seemed to be unrelated to a desire to obey or please them. Previous studies suggested that dog overimitation is rather related to affiliation. Dogs were found to overimitate their caregiver more than a stranger, and ‘perfect’ overimitators scored highly on caregiver-dog relationship tests (Huber et al., 2020, 2022). The present study further supports this affiliative hypothesis with its finding that dogs overimitated more often after the goal was already achieved – which was also a finding of Mackie and Huber (2023). These authors, alongside Taniguchi and Sanefuji (2021), argue that overimitation gains a more social goal once the instrumental goal (obtaining the reward) is completed. Particularly a disconnected irrelevant action (like our dot-touching or Taniguchi and Sanefuji’s box-tapping) had little chance to be misinterpreted as relevant, so obtaining the instrumental goal first and efficiently was a priority. Any overimitation afterwards would contain another goal: such as to be like or affiliate with the demonstrator. Together, these findings suggest that dogs, in our study, may have overimitated regardless of their caregiver’s attentional state due to such non-instrumental (social) goals.

For the relevant action, dogs showed sensitivity to their caregiver’s watchful state by copying the caregiver’s leftward push of the sliding door more than when their caregiver was inattentive. This result resembles those from obedience tasks regarding dogs’ sensitivity to attentional states. In obedience tasks, dogs were more likely to perform a ‘lie down’ command well if their commander (caregiver) was watching than if the commander left the room or turned around (Schwab & Huber, 2006; Virányi et al., 2004). Perhaps our dogs tried to follow the action cue from the caregiver's demonstration through faithful imitation (obtaining a perfect copying score) of the relevant action. And with the caregiver’s inattentiveness came no obligation to copy the same direction (left or right push) that the caregiver demonstrated. Taken together with the irrelevant action’s lower copying frequencies and its lack of sensitivity to attentional states, there are clear differing motivations behind these two kinds of copying; a strong, food prioritisation for the relevant action, and a secondary, ‘be-like’ motivation for the irrelevant action.

However, a full obedience-based interpretation would be assuming that the caregiver’s demonstration was viewed as some sort of non-verbal command. Our caregiver demonstration was designed to elicit the dog’s attention, with eye contact and name-calling if necessary. These caregiver behaviours typically occur during dog training contexts. So, if dogs viewed the demonstration as such a context, as a sort of command, then dogs who had reported training history should have been better at obtaining the reward (as seen in Marshall-Pescini et al., 2016). Yet, only six of the 13 reportedly trained dogs managed to do so (scored 3 + for relevant-action copying). Likewise, the irrelevant-action demonstration should also have been viewed as a command, but only six of the reportedly trained dogs were overimitators (scored 2 + for irrelevant-action copying). Training history alone, therefore, was not enough to support an obedience-based interpretation of dogs’ copying of the caregiver’s relevant action in this study. To do so would require further investigation, such as by having a much larger sample size, by including a forbidden food task (i.e., Call et al., 2003), or by manipulating a command-like strictness for the overimitation task’s demonstration. The inter-coder unreliability of our command-following behavioural scores from the dog-obedience test is one limitation of the present study.

Possibly both an intent to please *and* social signalling towards a watchful caregiver can explain why dogs copied their caregivers relevant action more accurately and approached the area of the dots (at least in in trial one) more often. Dogs respond more obediently when being watched in forbidden food/command tasks (e.g., Call et al., 2003; Schwab & Huber, 2006), which resembled their relevant-copying performance in this study. However, humans copy ‘better’ when being watched too. For humans, copying can be interpreted as a social signal towards the watcher. For example, adult humans precisely copied the height that their leader placed a block (in a game) if they were aware that their leader was watching them (Krishnan-Barman & Hamilton, 2019). By faithfully imitating their leader’s movements, the copiers sent a non-verbal communicative signal that they are paying close attention to their leader and maintaining their social connection. Mimicry, imitating someone’s gestures, etc., is also considered a social signal, and it can elicit positive opinions and prosocial dynamics towards the imitator (Farmer et al., 2018). One could argue that individuals send these social signals to *please* another person, which is something that dogs are well known for. So, we cannot exclude that dogs were closely imitating their (attentive) caregiver’s relevant actions to also please their caregivers in this study. As well as to be obedient, dogs may be using imitation as a social signal. If we consider the dog’s overimitation in terms of social signalling, their insensitivity to attentional states would mean that irrelevant copying wouldn’t have been used as this kind of social signal, at least from the overimitators in this study.

Our final findings for trials and the relevant-action behaviour replicated Mackie and Huber’s (2023), as dogs improved their copying of their caregiver’s relevant-action over trials. Dogs may have been gaining confidence to take the food reward in each trial from the increased awareness that it was permissible. Still, each trial offered dogs the opportunity to experience the directional push in the non-demonstrated direction, and often dogs would slide the door both ways while they were eating their food reward. So, the fact that dogs preferred to copy their caregiver’s leftward push over trials supports faithful imitation. Otherwise, dogs were approaching the irrelevant dots less each trial when being watched, and like in Johnston et al.’s (2017) study, these dogs (and dingoes) were likely learning that the action was inconsequential, or causally insignificant to the goal. However, around half of Johnston et al.’s dogs and dingoes were still performing the irrelevant (lever-pressing) action after four trials, and our dogs’ irrelevant-copying was unaffected by trial. Either (all) these persistent overimitators did not learn about the action’s irrelevance, even when given multiple chances to do so, or they had other motivations to overimitate.

In general, dog overimitation frequencies in the available experimental studies (Huber et al., 2018, 2020, 2022; Johnston et al., 2017; Mackie & Huber, 2023) are not as high or repetitive as in studies of human overimitation (i.e., Lyons et al., 2011). So rather than a universal phenomenon, like in humans, it seems that only certain kinds of dogs, with certain motivations, are engaging in overimitation of their closely bonded caregivers. By carefully balancing conditions for characteristics such as sex, breed and age, researchers might be missing key differences between dogs who overimitate and those who do not. For example, we counterbalanced our conditions for age yet still found an effect of age squared on the relevant action. Although literature on age and attachment is underexplored, it is suggested that a dog’s attachment to their caregiver changes with age over time (Rehn & Keeling, 2016). This may be a related factor to our finding. Future research on dog overimitation may wish to focus on such characteristics of dogs who *do* overimitate, and how contexts and experimental manipulations can affect these particular dogs’ motivation to overimitate. Perhaps non-overimitating dogs cannot, or care not to, ever become overimitators themselves.

## Conclusion

All in all, this study found that dogs were insensitive to their caregiver’s attentional state while copying irrelevant actions, but were sensitive to being watched while copying relevant actions and approaching the irrelevant dots. In particular, dogs copied their caregiver’s directional push more when they were being watched than when they were not, and approached the irrelevant dots less per trial when they were being watched. This sensitivity to attentional states aligns with studies on both dog obedience (in terms of command following) and human imitation behaviour. The dogs in this study who both overimitated and got the food reward, did so more often after obtaining the reward, as a kind of secondary goal. Our overimitation results indicate that following cues in a manner to please may not play a role in dog overimitation, instead, results support the affiliative hypothesis – that dogs overimitate because of their relationship and closeness with the demonstrator. In conclusion, the dogs in this study showed that they are sensitive to the attentional states of their caregiver, just not enough in the context of overimitation.

## Supplementary information

Below is the link to the electronic supplementary material.

Supplementary file1 (DOCX 39 KB)

## Data Availability

The datasets generated and analysed during the current study are available in the Open Science Framework repository at: https://osf.io/dkbsg/

## Code availability

The datasets analysed in R (R-Studio) during the current study are available in the Open Science Framework repository at: https://osf.io/dkbsg/

## Declarations

### Open practices statement

Data and coding materials for the experiments reported here are available in the Open Science Framework (OSF) repository at: https://osf.io/dkbsg/, and none of the experiments were preregistered.

## References

[CR1] Alexander, M. B., Friend, T., & Haug, L. (2011). Obedience training effects on search dog performance. *Applied Animal Behaviour Science,**132*(3), 152–159. 10.1016/j.applanim.2011.04.008

[CR2] Barr, D. J., Levy, R., Scheepers, C., & Tily, H. J. (2013). Random effects structure for confirmatory hypothesis testing: Keep it maximal. *Journal of Memory and Language,**68*(3), 255–278. 10.1016/j.jml.2012.11.001 10.1016/j.jml.2012.11.001 PMC3881361 24403724

[CR3] Call, J., Bräuer, J., Kaminski, J., & Tomasello, M. (2003). Domestic dogs (Canis familiaris) are sensitive to the attentional state of humans. *Journal of Comparative Psychology,**117*(3), 257–263. 10.1037/0735-7036.117.3.257 14498801 10.1037/0735-7036.117.3.257

[CR4] Dobson, A. J., & Barnett, A. G. (2018). An Introduction to Generalized Linear Models (4th ed.). Chapman and Hall/CRC. 10.1201/9781315182780

[CR5] Farmer, H., Ciaunica, A., de Hamilton, A. F., & C. (2018). The functions of imitative behaviour in humans. *Mind & Language,**33*(4), 378–396. 10.1111/mila.12189 30333677 10.1111/mila.12189 PMC6175014

[CR6] Fox, J., & Weisberg, S. (2019). Applied Regression 3E. In An R Companion to Applied Regression (Third Edition). Sage. https://socialsciences.mcmaster.ca/jfox/Books/Companion/appendices.html

[CR7] Fugazza, C., & Miklósi, Á. (2014). Should old dog trainers learn new tricks? The efficiency of the Do as I do method and shaping/clicker training method to train dogs. *Applied Animal Behaviour Science,**153*, 53–61. 10.1016/j.applanim.2014.01.009

[CR8] Fugazza, C., Moesta, A., Pogány, Á., & Miklósi, Á. (2018). Social learning from conspecifics and humans in dog puppies. Scientific Reports, 8(1), Article 1. 10.1038/s41598-018-27654-0 10.1038/s41598-018-27654-0 PMC6033932 29977034

[CR9] Fugazza, C., Pogány, Á., & Miklósi, Á. (2016). Do as I … Did! Long-term memory of imitative actions in dogs (Canis familiaris). *Animal Cognition,**19*(2), 263–269. 10.1007/s10071-015-0931-8 26498155 10.1007/s10071-015-0931-8

[CR10] Fugazza, C., Temesi, A., Coronas, R., Uccheddu, S., Gácsi, M., & Pogány, Á. (2023). Spontaneous action matching in dog puppies, kittens and wolf pups. *Scientific Reports,**13*(1), 2094. 10.1038/s41598-023-28959-5 36797322 10.1038/s41598-023-28959-5 PMC9935877

[CR11] Hoehl, S., Keupp, S., Schleihauf, H., McGuigan, N., Buttelmann, D., & Whiten, A. (2019). ‘Over-imitation’: A review and appraisal of a decade of research. *Developmental Review,**51*, 90–108. 10.1016/j.dr.2018.12.002

[CR12] Horner, V., & Whiten, A. (2005). Causal knowledge and imitation/emulation switching in chimpanzees (Pan troglodytes) and children (Homo sapiens). *Animal Cognition,**8*(3), 164–181. 10.1007/s10071-004-0239-6 15549502 10.1007/s10071-004-0239-6

[CR13] Huber, L. (1998). Movement imitation as faithful copying in the absence of insight. *Behavioral and Brain Sciences,**22*(5), 694.

[CR14] Huber, L., Kubala, D., & Cimarelli, G. (2022). Overimitation in Dogs: Is There a Link to the Quality of the Relationship with the Caregiver? Animals, 12(3). 10.3390/ani12030326 10.3390/ani12030326 PMC8833807 35158650

[CR15] Huber, L., Popovová, N., Riener, S., Salobir, K., & Cimarelli, G. (2018). Would dogs copy irrelevant actions from their human caregiver? *Learning and Behavior,**46*(4), 387–397. 10.3758/s13420-018-0336-z 29980941 10.3758/s13420-018-0336-z PMC6276069

[CR16] Huber, L., Range, F., & Virányi, Z. (2014). Dog Imitation and Its Possible Origins. In A. Horowitz (Ed.), Domestic Dog Cognition and Behavior: The Scientific Study of Canis familiaris (pp. 79–100). Springer. 10.1007/978-3-642-53994-7_4

[CR17] Huber, L., Range, F., Voelkl, B., Szucsich, A., Virányi, Z., & Miklosi, A. (2009). The evolution of imitation: What do the capacities of non-human animals tell us about the mechanisms of imitation? *Philosophical Transactions of the Royal Society B: Biological Sciences,**364*(1528), 2299–2309. 10.1098/rstb.2009.0060 10.1098/rstb.2009.0060 PMC2865073 19620102

[CR18] Huber, L., Salobir, K., Mundry, R., & Cimarelli, G. (2020). Selective overimitation in dogs. *Learning and Behavior,**48*(1), 113–123. 10.3758/s13420-019-00400-w 31975325 10.3758/s13420-019-00400-w

[CR19] Johnston, A. M., Holden, P. C., & Santos, L. R. (2017). Exploring the evolutionary origins of overimitation: A comparison across domesticated and non-domesticated canids. *Developmental Science,**20*(4), e12460. 10.1111/desc.12460 10.1111/desc.12460 27659592

[CR20] Kaminski, J., Pitsch, A., & Tomasello, M. (2013). Dogs steal in the dark. *Animal Cognition,**16*(3), 385–394. 10.1007/s10071-012-0579-6 23179109 10.1007/s10071-012-0579-6

[CR21] Krishnan-Barman, S., de Hamilton, A. F., & C. (2019). Adults imitate to send a social signal. *Cognition,**187*, 150–155. 10.1016/j.cognition.2019.03.007 30875661 10.1016/j.cognition.2019.03.007

[CR22] Kubinyi, E., Pongrácz, P., & Miklósi, Á. (2009). Dog as a model for studying conspecific and heterospecific social learning. *Journal of Veterinary Behavior,**4*(1), 31–41. 10.1016/j.jveb.2008.08.009

[CR23] Kupán, K., Miklósi, Á., Gergely, G., & Topál, J. (2011). Why do dogs (Canis familiaris) select the empty container in an observational learning task? *Animal Cognition,**14*(2), 259–268. 10.1007/s10071-010-0359-0 21140185 10.1007/s10071-010-0359-0

[CR24] Lonardo, L., Völter, C. J., Lamm, C., & Huber, L. (2021). Dogs follow human misleading suggestions more often when the informant has a false belief. *Proceedings of the Royal Society B: Biological Sciences,**288*(1955), 20210906. 10.1098/rspb.2021.0906 10.1098/rspb.2021.0906 PMC8292766 34284633

[CR25] Lyons, D. E., Damrosch, D. H., Lin, J. K., Macris, D. M., & Keil, F. C. (2011). The scope and limits of overimitation in the transmission of artefact culture. *Philosophical Transactions of the Royal Society B: Biological Sciences,**366*(1567), 1158–1167. 10.1098/rstb.2010.0335 10.1098/rstb.2010.0335 PMC3049096 21357238

[CR26] Lyons, D. E., Young, A. G., & Keil, F. C. (2007). The hidden structure of overimitation. *Proceedings of the National Academy of Sciences,**104*(50), 19751–19756. 10.1073/pnas.0704452104 10.1073/pnas.0704452104 PMC2148370 18056814

[CR27] Mackie, L., & Huber, L. (2023). Socially priming dogs in an overimitation task. Frontiers in Psychology, 14. https://www.frontiersin.org/articles/. 10.3389/fpsyg.2023.1063132 10.3389/fpsyg.2023.1063132 PMC9982082 36874835

[CR28] MacLean, E. L., Herrmann, E., Suchindran, S., & Hare, B. (2017). Individual differences in cooperative communicative skills are more similar between dogs and humans than chimpanzees. *Animal Behaviour,**126*, 41–51. 10.1016/j.anbehav.2017.01.005

[CR29] Maoz, I., Zubedat, S., Dolev, T., Aga-Mizrachi, S., Bloch, B., Michaeli, Y., Eshed, Y., Grinstein, D., & Avital, A. (2021). Dog training alleviates PTSD symptomatology by emotional and attentional regulation. *European Journal of Psychotraumatology,**12*(1), 1995264. 10.1080/20008198.2021.1995264 34868486 10.1080/20008198.2021.1995264 PMC8635621

[CR30] Marsh, L. E., Ropar, D., de Hamilton, A. F., & C. (2019). Are you watching me? The role of audience and object novelty in overimitation. *Journal of Experimental Child Psychology,**180*, 123–130. 10.1016/j.jecp.2018.12.010 30655097 10.1016/j.jecp.2018.12.010

[CR31] Marshall-Pescini, S., Frazzi, C., & Valsecchi, P. (2016). The effect of training and breed group on problem-solving behaviours in dogs. *Animal Cognition,**19*(3), 571–579. 10.1007/s10071-016-0960-y 26861484 10.1007/s10071-016-0960-y

[CR32] Nakagawa, S., & Schielzeth, H. (2013). A general and simple method for obtaining R2 from generalized linear mixed-effects models. *Methods in Ecology and Evolution,**4*(2), 133–142.

[CR33] Nieuwenhuis, R., Grotenhuis, M. te, & Pelzer, B. (2012). influence.ME: Tools for Detecting Influential Data in Mixed Effects Models. The R Journal, 4(2), 38–47.

[CR34] Palmer, R., & Custance, D. (2008). A counterbalanced version of Ainsworth’s Strange Situation Procedure reveals secure-base effects in dog–human relationships. *Applied Animal Behaviour Science,**109*(2), 306–319. 10.1016/j.applanim.2007.04.002

[CR35] Pelgrim, M. H., Espinosa, J., Tecwyn, E. C., Marton, S. M., Johnston, A., & Buchsbaum, D. (2021). What’s the point? Domestic dogs’ sensitivity to the accuracy of human informants. *Animal Cognition,**24*(2), 281–297. 10.1007/s10071-021-01493-5 33675439 10.1007/s10071-021-01493-5 PMC7936605

[CR36] R Core Team. (2022). R: The R Project for Statistical Computing. https://www.r-project.org/

[CR37] Range, F., Huber, L., & Heyes, C. (2010). Automatic imitation in dogs. *Proceedings of the Royal Society B: Biological Sciences,**278*(1703), 211–217. 10.1098/rspb.2010.1142 10.1098/rspb.2010.1142 PMC3013390 20667875

[CR38] Rehn, T., & Keeling, L. J. (2016). Measuring dog-owner relationships: Crossing boundaries between animal behaviour and human psychology. *Applied Animal Behaviour Science,**183*, 1–9. 10.1016/j.applanim.2016.07.003

[CR39] Schielzeth, H. (2010). Simple means to improve the interpretability of regression coefficients. *Methods in Ecology and Evolution,**1*(2), 103–113. 10.1111/j.2041-210X.2010.00012.x

[CR40] Schielzeth, H., & Forstmeier, W. (2009). Conclusions beyond support: Overconfident estimates in mixed models. *Behavioral Ecology,**20*(2), 416–420. 10.1093/beheco/arn145 19461866 10.1093/beheco/arn145 PMC2657178

[CR41] Schleihauf, H., & Hoehl, S. (2021). Evidence for a dual-process account of over-imitation: Children imitate anti- and prosocial models equally, but prefer prosocial models once they become aware of multiple solutions to a task. *PLoS ONE,**16*(9), e0256614. 10.1371/journal.pone.0256614 34529702 10.1371/journal.pone.0256614 PMC8445421

[CR42] Schwab, C., & Huber, L. (2006). Obey or Not Obey? Dogs (Canis familiaris) Behave Differently in Response to Attentional States of Their Owners. *Journal of Comparative Psychology,**120*(3), 169–175. 10.1037/0735-7036.120.3.169 16893253 10.1037/0735-7036.120.3.169

[CR43] Stengelin, R., Hepach, R., & Haun, D. B. M. (2019). Being observed increases overimitation in three diverse cultures. *Developmental Psychology,**55*(12), 2630–2636. 10.1037/dev0000832 31599607 10.1037/dev0000832

[CR44] Taniguchi, Y., & Sanefuji, W. (2021). Irrelevant actions, goal demotion and explicit instruction: A study of overimitation. *Infant and Child Development,**30*(4), e2227. 10.1002/ICD.2227

[CR45] Topál, J., Byrne, R. W., Miklósi, Á., & Csányi, V. (2006). Reproducing human actions and action sequences: “Do as I Do!” in a dog. *Animal Cognition,**9*(4), 355–367. 10.1007/s10071-006-0051-6 17024511 10.1007/s10071-006-0051-6

[CR46] Underwood, A. J. (1997). *Experiments in ecology: Their logical design and interpretation using analysis of variance*. Cambridge University Press.

[CR47] Virányi, Z., Topál, J., Gácsi, M., Miklósi, Á., & Csányi, V. (2004). Dogs respond appropriately to cues of humans’ attentional focus. *Behavioural Processes,**66*(2), 161–172. 10.1016/j.beproc.2004.01.012 15110918 10.1016/j.beproc.2004.01.012

[CR48] Völter, C. J., Lonardo, L., Steinmann, M. G. G. M., Ramos, C. F., Gerwisch, K., Schranz, M.-T., Dobernig, I., & Huber, L. (2023). Unwilling or unable? Using three-dimensional tracking to evaluate dogs’ reactions to differing human intentions. *Proceedings of the Royal Society B: Biological Sciences,**290*(1991), 20221621. 10.1098/rspb.2022.1621 10.1098/rspb.2022.1621 PMC9874264 36695031

[CR49] Whiten, A., McGuigan, N., Marshall-Pescini, S., & Hopper, L. M. (2009). Emulation, imitation, over-imitation and the scope of culture for child and chimpanzee. *Philosophical Transactions of the Royal Society B: Biological Sciences,**364*(1528), 2417–2428. 10.1098/rstb.2009.0069 10.1098/rstb.2009.0069 PMC2865074 19620112

