# Supplementary material for: Watched or not: Overimitation in dogs under different attentional states
Source: Learn Behav. 2024 Sep 26;53(2):171–82. doi: 10.3758/s13420-024-00635-2 (PMC12092473; doi:10.3758/s13420-024-00635-2)
Supplement: Supplementary file 1 — Supplementary file1 (DOCX 39 KB) [file 13420_2024_635_MOESM1_ESM.docx]

**Watched or not: Overimitation in dogs under different attentional states**

Louise Mackie^1*^, Jeanne Trehorel^1^, and Ludwig Huber^1^

^email:^ [^Louise.Mackie@vetmeduni.ac.at^](mailto:Louise.Mackie@vetmeduni.ac.at) [^jeannetrehorel@gmail.com^](mailto:jeannetrehorel@gmail.com) [^Ludwig.Huber@vetmeduni.ac.at^](mailto:Ludwig.Huber@vetmeduni.ac.at)

^orcid ID: 0009-0004-6834-2987 0000-0002-0217-136X^

^1^Comparative Cognition, Messerli Research Institute, Department of Interdisciplinary Life Sciences, University of Veterinary Medicine Vienna, Vienna, Austria

***Correspondence:**Louise Mackie
[Louise.Mackie@vetmeduni.ac.at](mailto:Louise.Mackie@vetmeduni.ac.at)

University of Veterinary Medicine Vienna, Veterinärplatz 1, 1210 Wien, Austria

**Keywords**: domestic dogs, attentional states, copying, overimitation, social learning

**Appendix**

| **Appendix A.** The behavioural scoring descriptions for the command-following scores from the obedience test. | | |
| --- | --- | --- |
| **Command-Following Score** | **Score Description** | **Command Description** |
| 0 | no commands followed | **-** |
| 1 | one successful command | Sit |
| 2 | two successful commands | Stay for 20 seconds |
| 3 | three successful commands | Recall to caregiver |
| 4 | four successful commands | Return to experimenter |
| 5 | all commands successful | Sit again next to experimenter |

| **Appendix B.** Results of the non-significant ordinal mixed model for irrelevant-action scores (0-4). | | | | | | | | | |
| --- | --- | --- | --- | --- | --- | --- | --- | --- | --- |
| **Term (effect)** | **Model Estimate** | **Std. Error** | **lower cl** | **upper cl** | ***χ2*** | ***df*** | ***P-value*** | **min** | **max** |
| 0\|1 | -0.503 | 0.432 | -1.399 | 0.363 |  |  | ^(3)^ | -0.716 | -0.368 |
| 1\|2 | 2.227 | 0.469 | 1.385 | 3.271 |  |  | ^(3)^ | 2.001 | 2.430 |
| 2\|3 | 4.682 | 0.684 | 3.508 | 6.221 |  |  | ^(3)^ | 4.440 | 5.149 |
| 3\|4 | 4.982 | 0.744 | 3.773 | 6.727 |  |  | ^(3)^ | 4.739 | 5.574 |
| ^(1)^Condition_watching | 0.104 | 0.403 | -0.713 | 0.974 | 0.066 | 1 | 0.797 | -0.076 | 0.255 |
| **^(2)^z.trial** | **-0.322** | **0.133** | **-0.577** | **-0.078** | **6.084** | **1** | **0.014** | **-0.366** | **-0.293** |
| ^(2)^z.age | -0.190 | 0.269 | -0.742 | 0.340 | 0.499 | 1 | 0.480 | -0.302 | -0.105 |
| ^(2)^I(z.age^2) | -0.046 | 0.167 | -0.400 | 0.242 | 0.075 | 1 | 0.784 | -0.084 | 0.017 |
| ^(1)^sexM | -0.095 | 0.383 | -0.880 | 0.642 | 0.061 | 1 | 0.805 | -0.257 | 0.064 |
| **Note:** cl represents the confidence interval limit, χ^2^ represents the chi-square statistic, df represents degrees of freedom, p-value indicates the significance level (bold rows are significant; p<.05), and min and max are the minimum and maximum model stability estimates.  (1) Condition and sex were dummy coded with ‘Condition_turned-away’ and ‘female’ being the reference categories respectively. | | | | | | | | | |
| (2) Trial number and age were z-transformed to a mean of zero and a standard deviation of one, mean (sd) of trial number was 2.49 (1.12), mean (sd) of age was 3.88 (2.83) | | | | | | | | | |
| (3) not indicated because of having a very limited interpretation | | | | | | | | | |

| **Appendix D.** The means of approaching the dots (yes/no) per condition and trial. | | | |
| --- | --- | --- | --- |
| **Trial** | **Condition** | **^(1)^Mean** | **Standard Error** |
| 1 | turned-away | 0.6 | 0.091 |
| 1 | watching | 0.788 | 0.072 |
| 2 | turned-away | 0.7 | 0.085 |
| 2 | watching | 0.545 | 0.088 |
| 3 | turned-away | 0.567 | 0.092 |
| 3 | watching | 0.606 | 0.086 |
| 4 | turned-away | 0.586 | 0.093 |
| 4 | watching | 0.364 | 0.085 |
| **Note:** The purpose of obtaining these means was to plot a visual representation of the binomial mixed model’s interaction for Figure 3  ^(1)^Means were calculated from the binary approach scores (0 for no, 1 for yes). | | | |

| **Appendix C.** Results of the non-significant binomial mixed model for overimitation behaviour (yes/no). | | | | | | | |
| --- | --- | --- | --- | --- | --- | --- | --- |
| **Term (effect)** | **Model Estimate** | **Std. Error** | **Z value** | **df** | **P-value** | **min** | **max** |
| (intercept) | -2.87 | 0.609 | -4.71 | 1 | <0.001 | -3.281 | -2.57 |
| Condition_watching | 0.436 | 0.587 | 0.742 | 1 | 0.458 | 0.09 | 0.794 |
| ^(1)^z.trial | 0.132 | 0.299 | 0.442 | 1 | 0.659 | -0.031 | 0.329 |
| ^(1)^z.age | -0.522 | 0.394 | -1.327 | 1 | 0.185 | -0.785 | -0.408 |
| ^(1)^I(z.age^2) | 0.254 | 0.226 | 1.126 | 1 | 0.26 | 0.199 | 0.369 |
| Condition_watching*z.trial | -0.297 | 0.405 | -0.733 | 1 | 0.463 | -0.495 | -0.134 |
| **Note:** z-value represents the z statistic, df represents degrees of freedom, p-value indicates the significance level (bold rows are significant; p<.05), and min and max are the minimum and maximum model stability estimates.   1. Trial number and age were z-transformed to a mean of zero and a standard deviation of one, mean (sd) of trial number was 2.49 (1.12), mean (sd) of age was 3.96 (2.89) | | | | | | | |
